# Study on the differences of gene expression between pear and apple wild cultivation materials based on RNA-seq technique

**DOI:** 10.1186/s12870-021-03051-0

**Published:** 2021-06-04

**Authors:** Huangwei Zhang, Meng Li, Min Kong, Jim M. Dunwell, Yuyan Zhang, Chao Yue, Juyou Wu, Shaoling Zhang

**Affiliations:** 1 College of Horticulture, Nanjing Agricultural University, Nanjing, 210095 China; 2 College of Agro-Grassland Science, Nanjing Agricultural University, Nanjing, 210095 China; 3 School of Agriculture, Policy and Development, University of Reading, Earley Gate, Reading, UK; 4 Institute of Pomology, Jiangsu Academy of Agricultural Sciences/Jiangsu Key Laboratory of Horticultural Crop Genetic Improvement, Nanjing, 210014 Jiangsu China; 5 China Tobacco Jiangsu Industrial Co., Ltd, Nanjing, 210019 China

**Keywords:** Pear, Apple, Wild cultivar, Differential expression, Domestication, RNA-seq

## Abstract

**Background:**

Pears and apples are both perennial deciduous trees of the *Rosaceae* family, and both are important economic fruit trees worldwide. The emergence of many varieties in the market has been mostly domesticated from wild to cultivated and regulated by the differential expression of genes. However, the molecular process and pathways underlying this phenomenon remain unclear. Four typical wild and cultivar pear and apple trees at three developmental stages were used in our study to investigate the molecular process at the transcriptome level.

**Result:**

Physiological observations indicated the obvious differences of size, weight, sugar acid content and peel color in wild and cultivar fruit among each developmental stage. Using next-generation sequencing based RNA-seq expression profiling technology, we produced a transcriptome in procession of a large fraction of annotated pear and apple genes, and provided a molecular basis underlying the phenomenon of wild and cultivar fruit tree differences. 5921 and 5744 differential expression genes were identified in pear and apple at three developmental stages respectively. We performed temporal and spatial differential gene expression profiling in developing fruits. Several key pathways such as signal transduction, photosynthesis, translation and many metabolisms were identified as involved in the differentiation of wild and cultivar fruits.

**Conclusion:**

In this study, we reported on the next-generation sequencing study of the temporal and spatial mRNA expression profiling of pear and apple fruit trees. Also, we demonstrated that the integrated analysis of pear and apple transcriptome, which strongly revealed the consistent process of domestication in *Rosaceae* fruit trees. The results will be great influence to the improvement of cultivar species and the utilization of wild resources.

**Supplementary Information:**

The online version contains supplementary material available at 10.1186/s12870-021-03051-0.

## Background

Pear and apple trees belong to the deciduous perennial trees of *Malinae* subtribe within *Rosaceae* (*Pyrus*) family [1]. Both of them are important economic fruits worldwide, and China is the main producer and consumer (FAO, 2020). Pear originated from the wild pear ‘Douli’ (*P. calleryana* Dence) southwest China, which has a cultivation history of more than 3000 years in China [2, 3]. Cultivar pears trees include *P. bretschneideri*, *P. pyrifolia*, *P. ussuriensis*, *P. sinkiangensis*, and *P. communis* system [4], while typical wild pears include Du pear (*P. betulifolia* Bunge), Chuan pear (*P. pashia* Buch), Shan pear (*P. ussuriensis* Maxim) and Ma pear (*P. serrulata* Rehd) [5]. Wild apple species originated 3 million years ago, and the fossil remains of apple fruits in the Anatolia Peninsula in Western Asia can be traced back to about 6500 BC [6]. Numbers of studies believed that the wild apple specie in Central Asia was the ancestors of modern cultivated apples, namely *M. sieversii* (Ledeb.) Roem [6, 7].

Fruit tree domestication is a process of adapting wild fruit trees to a new environment by changing their genetic characteristics [8]. Generally, wild species have smaller fruits, oblate in shape, with low sugar content, high acid content, rough pericarp, small leaves and short petiole [9, 10]. According to individual preferences, people often choose fruit with a large size, bright color and good flavor, all these characteristics are required if a cultivar is to be widely planted. Therefore, the quality of fruit such as fruit size, sugar and acid content, peel color, and fruit firmness has always been a focus of research [11–13]. With the completion of several genome sequences in pear and apple [14–17], research on fruit evolution and quality has expanded greatly. Some related genes such as *miR172g*, *MdSWEET9b, MdSWEET15a* and *MYB* have been identified in fruit trees [7, 18, 19]. Despite a set of draft genome, SNP and QTL in pear and apple [17, 20–22], the molecular process underlying huge changes between wild and cultivar fruit remain unclear.

Measurements of mRNA expression levels, clarity of the regulatory relationships between them are critical to understanding many pathways and biological systems. With the advent of second-generation sequencing-based technologies of RNA-seq, it is possible to measure a genome wide dynamic range of expression in an unbiased manner [23]. RNA-seq technology have a high sensitivity and reproducibility and will undoubtedly lead to novel insights into plant development and response [24].

In the present study, the wild pear ‘Douli’ (*P. calleryana* Dence), wild apple ‘Xifuhaitang’ (*M. micromalus* Makino) and the cultivar pear ‘Dangshansuli’ (*P. bretschneideri* Rehd), cultivar apple ‘Golden Delicious’ (*M. domestica*) were selected as typical materials for wild and cultivar fruit tree [2, 14, 25, 26]. To comprehensively understand the molecular pathway underlying the huge changes of wild and cultivar at the whole genome level, we have studied the differently expressed genes in different development stages both in pear and apple using RNA-seq technology. The results will further explore the regulation pathways of pear and apple domestication, especially the differences in fruit size, fruit flavor, peel color and fruit resistance, which will be great influence to the improvement of cultivated species and the utilization of wild resources.

## Results

### Transcript RNA sequence dataset of pear and apple libraries

**Fig. 1:**
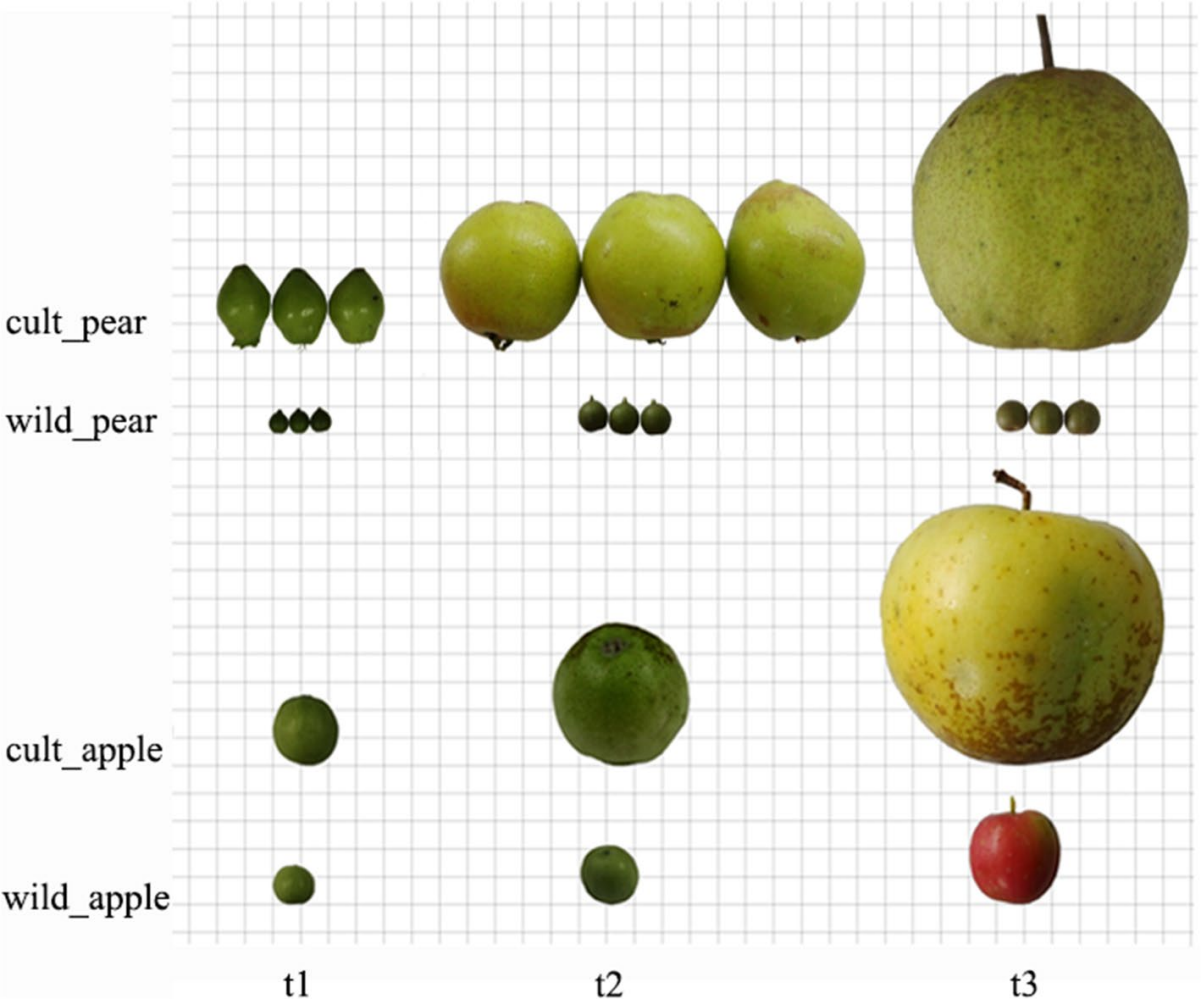
Plant materials at three developmental stages. t1, t2 and t3 represent the young fruit stage, the expansion stage and the mature stage respectively. Each square represents 1 cm

In this study, RNA of different types of materials (including two species and three developmental stages) were pooled to provide a broad gene library associated with fruit growth and finally sixteen libraries were generated including the fruits and leaves (Fig. 1, Table 1). A total of 14,937,456—30,370,082 reads were obtained from eight libraries of pear, 57.8%—71.8% of which could be mapped to ‘Dangshansuli’ reference genome and concordant-pairs value is 50.5%—66.2% [14]. A total of 16,219,542—34,807,619 reads were obtained from eight libraries of apple, 61.0%—76.3% of which could be mapped to the ‘Golden Delicious’ genome and concordant-pairs value is 54.2%—72.5% [17]. We obtained 84,055 transcripts and 40,402 genes with N50 length 2306 bp and median length 1643. 95 bp in pear; we obtained 95,600 transcripts and 56,781 genes with N50 length 2117 bp and median length 1470.5 bp in apple (Table 2).

**Table 1:** Stages and abbreviations of the pear and apple libraries

Pear		Apple	
CP_t1	May 6^th^, 2014/ cultivar, young fruit stage fruit	CA_t1	May 6^th^, 2014/ cultivar, young stage fruit
CP_t2	June 30^th^, 2014/ cultivar, expansion stage fruit	CA_t2	June 6^th^, 2014/ cultivar, expansion stage fruit
CP_t3	September 6^th^, 2014/ cultivar, mature stage fruit	CA_t3	September 17^th^, 2014/ cultivar, mature stage fruit
CP_leaf	September 6^th^, 2014/ cultivar, leaves and branches	CA_leaf	October 30^th^, 2014/ cultivar, leaves and branches
WP_t1	May 6^th^, 2014/ wild, young fruit stage fruit	WA_t1	May 6^th^, 2014/ wild, young fruit stage fruit
WP_t2	June 30^th^, 2014/ wild, expansion stage fruit	WA_t2	June 6^th^, 2014/ wild, expansion stage fruit
WP_t3	September 6^th^, 2014/ wild, mature stage fruit	WA_t3	September 17^th^, 2014/ wild, mature stage fruit
WP_leaf	September 6^th^, 2014/ wild, leaves and branches	WA_leaf	October 30^th^, 2014/ wild, leaves and branches

**Table 2:** Summary of mapping reads

Sample id	Total pairs	Mapped reads (%)	Concordant pairs (%)	Sample id	Total pairs	Mapped reads (%)	Concordant pairs
CP_t1	14,937,456	70.6	65.8	CA_t1	16,219,542	76.1%	71.8%
CP_t2	17,645,457	69.3	64.2	CA_t2	30,189,007	76.3%	72.5%
CP_t3	30,034,015	71	66.2	CA_t3	34,807,619	72.7%	68.7%
CP_leaf	24,157,305	62.5	56.4	CA_leaf	22,513,062	72.3%	67.9%
WP_t1	19,665,537	60.9	52.6	WA_t1	17,375,339	63.1%	55.7%
WP_t2	29,976,758	61.1	52.9	WA_t2	29,481,030	64.0%	57.4%
WP_t3	30,370,082	62.3	53.2	WA_t3	30,772,914	65.0%	58.4%
WP_leaf	23,136,777	57.8	50.5	WA_leaf	25,821,118	61.0%	54.2%

### Physiological index variation at developmental stages of pear and apple fruits

**Fig. 2:**
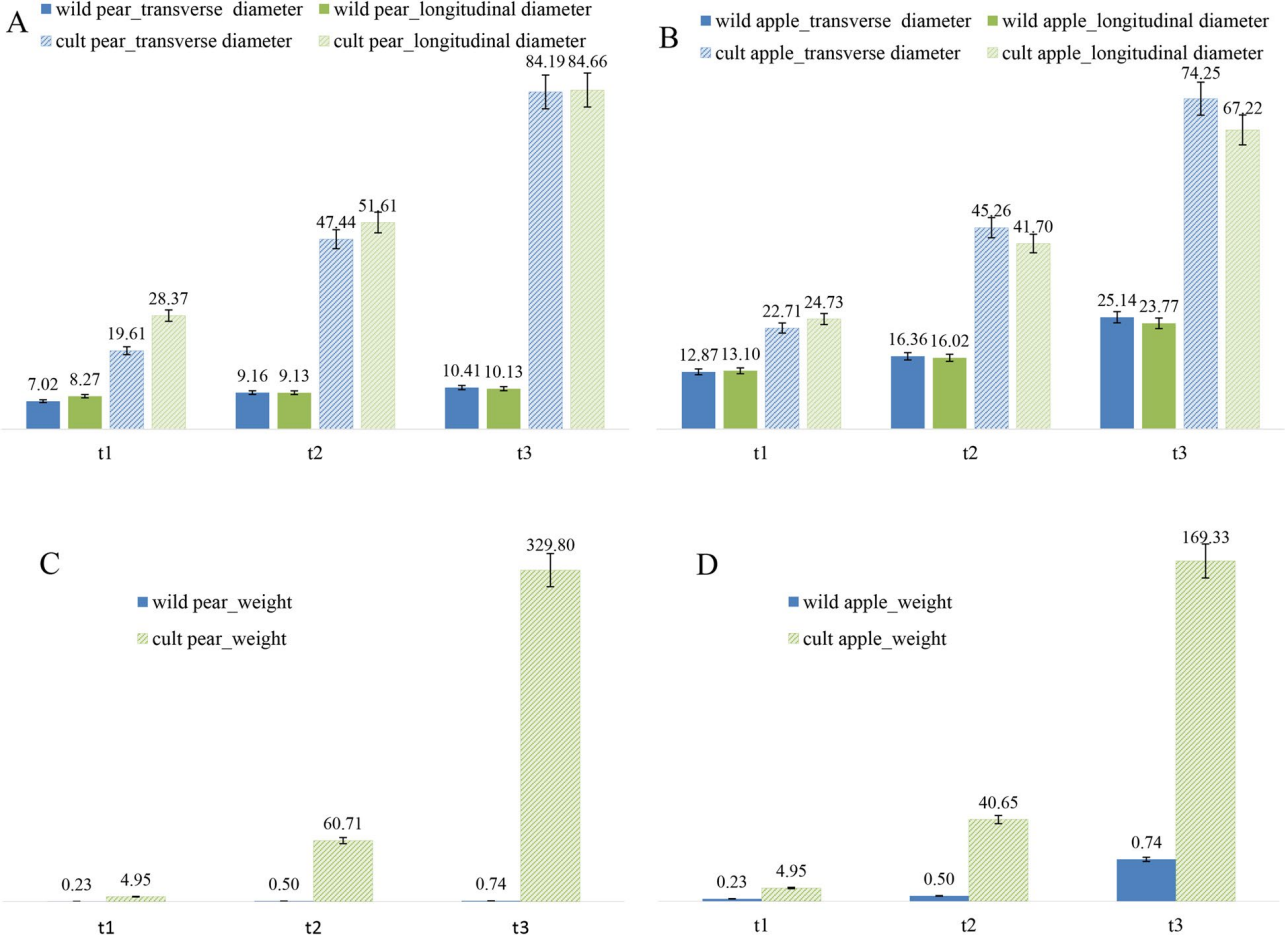
Fruit size and single fruit weight of wild and cultivar fruits at three stages. A&B: fruit size, ordinate represents transverse and longitudinal diameter value (mm), blue represents transverse diameter, green represents longitudinal diameter. C&D: fruit weight, ordinate represents the value of fruit weight (g); solid represents the wild apple; slant represents the cultivated apple; abscissa t1, t2, t3 represents the young fruit stage, expansion stage and mature stage respectively

To better understand the physiological variation among growth, transverse and longitudinal diameter, single fruit weight and sugar acid content from the developmental stages were observed (Fig. 2, Table 3). The transverse and vertical diameter of wild pear at maturity were 1.46 and 1.23 times of that at young fruit stage, respectively (Fig. 2A). The growth rate of wild pear was slow and the fruit size had no obvious change during the fruit development process. The transverse and vertical diameter of cultivar pear at maturity stage were 4.3 and 2.9 times of that at young fruit stage, respectively. The lateral growth rate of cultivar pear is higher than that of longitudinal growth, and the growth rate of fruit size is much faster than that of wild. The transverse and longitudinal diameters of wild apple at the young fruit stage were 12.87 mm and 13.1 mm respectively, while at the mature stage they were 25.14 mm and 23.77 mm, which were about 1.95 and 1.48 times that at young fruit stage (Fig. 2B). The fruit grew slowly and the size did not change significantly. In contrast, the transverse and longitudinal diameters of cultivar apple at maturity were 74.25 mm and 67.22 mm respectively, which were about 3.27 and 2.72 times that at the young fruit stage. The weight at maturity stage of cultivar pear is about 447 times of that of wild (Fig. 2C). The weight of cultivar apple at maturity stage was 8.12 times that of wild (Fig. 2D). There are obvious differences in volume and weight between the wild and cultivar, with both fruit characteristics being greater in the cultivar.

**Table 3:** Sugar and acid contents of wild and cultivar fruits at three stages

Content (mg/g)	CP_t1	CP_t2	CP_t3	WP_t1	WP_t2	WP_t3	CA_t1	CA_t2	CA_t3	WA_t1	WA_t2	WA_t3
Sorbitol	18.20	13.09	8.67	5.88	1.35	7.02	3.77	3.47	5.11	4.73	6.27	5.83
Fructose	4.57	12.45	26.24	0.66	-	5.27	20.09	36.72	73.27	8.24	17.48	28.67
Glucose	1.44	3.32	7.41	-	-	4.54	7.05	13.71	27.12	4.77	12.09	14.15
Sucrose	1.16	1.28	2.18	0.38	0.40	3.83	3.62	5.75	25.73	2.81	9.98	12.80
Quinic acid	10.68	1.22	0.59	2.74	1.23	1.86	2.18	0.73	0.23	3.35	1.46	0.45
Citric acid	-	-	-	-	0.07	4.80	-	-	-	7.91	-	8.18
Malic acid	1.47	0.96	0.84	2.69	0.90	2.59	9.62	6.66	1.97	13.87	10.91	6.36
Oxalic acid	0.39	0.17	0.12	0.27	0.16	0.26	0.25	0.10	0.03	0.11	0.09	0.001
Shikimic acid	0.27	0.28	-	0.19	0.02	-	0.05	0.03	-	0.06	0.03	-

The soluble sugars in pear and apple mainly includes sorbitol, fructose, glucose and sucrose [27]. The organic acids are mainly include quinic acid, citric acid, malic acid, oxalic acid and shikimic acid (Table 3) [28]. The total sugar content of cultivar pear at maturity reached 43.6 $\mathrm{mg}\bullet \mathrm{g}$^−1^, and 20.66 $\mathrm{mg}\bullet \mathrm{g}$^−1^ in wild, which showed that the total sugar content of cultivar pear was higher than that of wild. The content of fructose in cultivar pear was 26.24 $\mathrm{mg}\bullet \mathrm{g}$^−1^ at maturity stage, about 13 times that of sucrose. The contents of glucose, fructose and sucrose increased with fruit development, and were positively correlated with total sugar content. There was a negative correlation between sorbitol and total sugar content. The content of citric acid in wild pear is higher than that of other acids. The content of organic acids in wild pear maturity stage is higher than that in cultivar.

The fructose content was higher than other sugars and in cultivar apple at maturity was about 2.6 times of that in wild apple. The total sugar content at maturity of cultivar apple reached 131.23 $\mathrm{mg}\bullet \mathrm{g}$^−1^, and wild apple reached 61.45 $\mathrm{mg}\bullet \mathrm{g}$^−1^, which showed that the total sugar content in cultivar apple was significantly higher than in wild. The contents of glucose, fructose and sucrose increased during fruit development, and were positively correlated with total sugar content. The content of citric acid and malic acid were higher than the other acids. The highest acid content of wild apple at maturity was citric acid 8.18 $\mathrm{mg}\bullet \mathrm{g}$^−1^, followed by malic acid content of 6.36 $\mathrm{mg}\bullet \mathrm{g}$^−1^. In contrast to sugar content, the organic acid content decreased during fruit maturation. The total acid content in wild was higher than in cultivar apple.

### Identification of differentially expressed transcripts and genes in wild and cultivar fruit at three stages

**Fig. 3:**
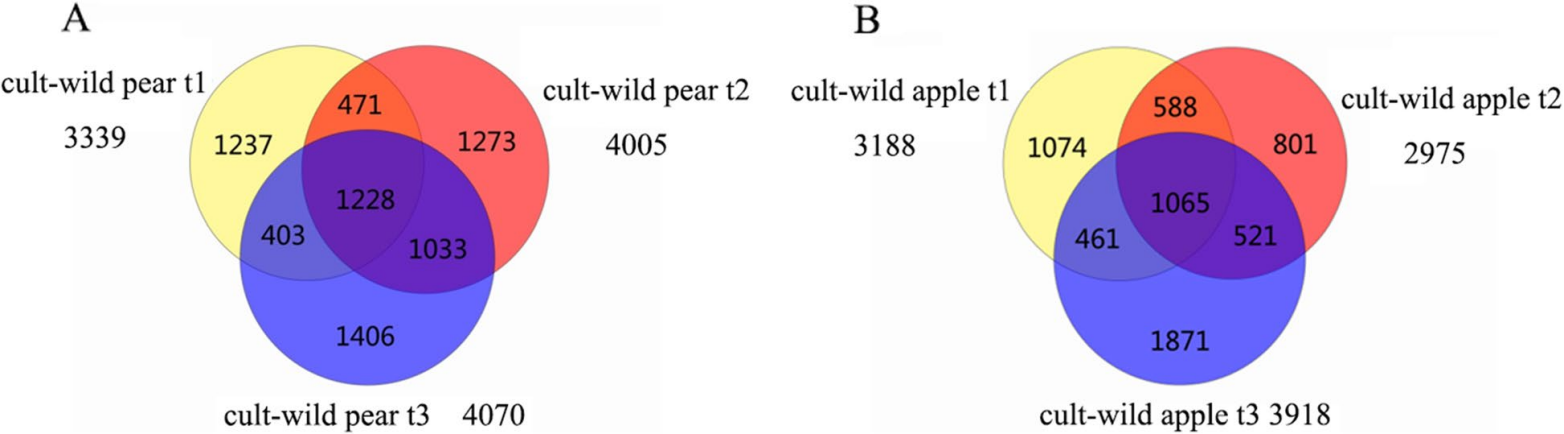
The number of differentially expressed transcripts of wild and cultivar samples at three stages. t1, t2 and t3 represent the young fruit stage, the expansion stage and the mature stage respectively

According to *P* < 0.001 and |Fold change|> 2, the significant differential expressed transcripts were identified (Fig. 3). There were 3339, 4005 and 4070 differential expressed transcripts of the two pear varieties at young fruit stage, expanding stage and mature stage, respectively (Fig. 3A). 7051 transcripts were left after removing duplicate transcripts in all three stages, which corresponded to 5921 genes. There are 1228 transcripts that were differentially expressed at all stages; this corresponds to 1068 genes. There were 2261 (1228 + 1033) differential expressed transcripts existing simultaneously at the expanding stage and the mature stage, and 1699 (1228 + 471) and 1631 (1228 + 403) at the young fruit stage and expanding stage, the young fruit stage and mature stage, respectively (Fig. 3A).

The numbers of differential expression transcripts were 3188, 2975, and 3918 at apple young fruit stage, expansion stage and mature stage respectively (Fig. 3B). The intersection and union of the three sets were 1065 and 6381 respectively, corresponding to 969 and 5744 genes respectively. The common differentially expressed transcripts between expansion and mature stage, young and expansion stage, young and mature stage were 1586, 1653 and 1526 respectively. The independent differentially expressed transcripts at young fruit stage, expansion stage and mature stage were 1074, 801 and 1871 respectively; these were only expressed in a single stage.

### Expression trend analysis of differential expression genes at three periods

**Fig. 4:**
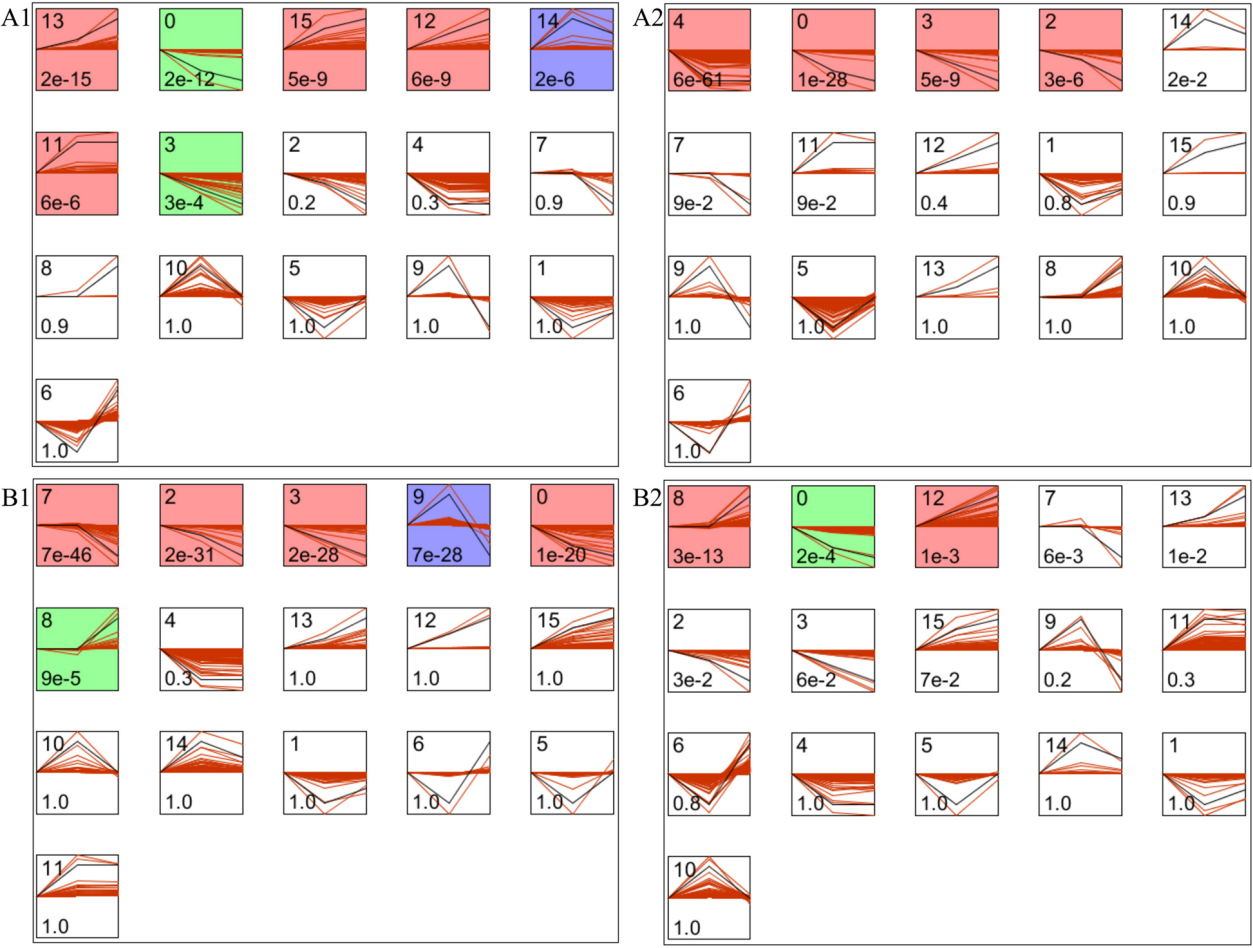
Expression trend profiles of differentially expressed genes at three stages of cultivar pear (**A1**), wild pear (**A2**), cultivar apple (**B1**) and wild apple (**B2**) respectively. The number of differential expression genes in pear and apple was 5921, 5744 respectively. Each box represents a different expression profile, colored profiles have a statistically significant number of genes assigned with *P* < 0.05, the upper left corner of the digital is profile ID, the lower right corner represents *P* value

5921 and 5744 different expression genes were further clustered using Short Time-series Expression Miner (STEM) to analyze the expression trend in pear and apple respectively [29]. It identified 16 model expression profiles both in pear and apple (Fig. 4). Colored profiles are statistically significant assigned with *P* < 0.05. Among the 7 colored profiles of cultivar pear, profile 13 (397 genes), 15 (239 genes), 12 (208 genes) and 11 (319 genes) were up-regulated, profile 0 (381 genes) and 3 (179 genes) were down-regulated and profile 14 (484 genes) was first up-regulated and then down-regulated (Fig. 4A1). Four profiles (2007 genes) with significantly colored in wild pear and all of them were down-regulated (Fig. 4A2).

There were 6 colored profiles are significantly at three stages of cultivar apple (Fig. 4B1) and 3 profiles in wild apple (Fig. 4B2). In cultivar apple, profile 7 (539 genes), 2 (345 genes), 3 (298 genes) and 0 (382 genes) were down-regulated, profile 8 (692 genes) was up-regulated, and profile 9 (623 genes) was up-regulated at the beginning and then down-regulated (Fig. 4B1). In wild apple, profile 8 (726 genes) and profile 12 (174 genes) were up-regulated, and profile 0 (299 genes) was down-regulated (Fig. 4B2).

### Go functional annotation enrichment analysis of differential expression genes

**Fig. 5:**
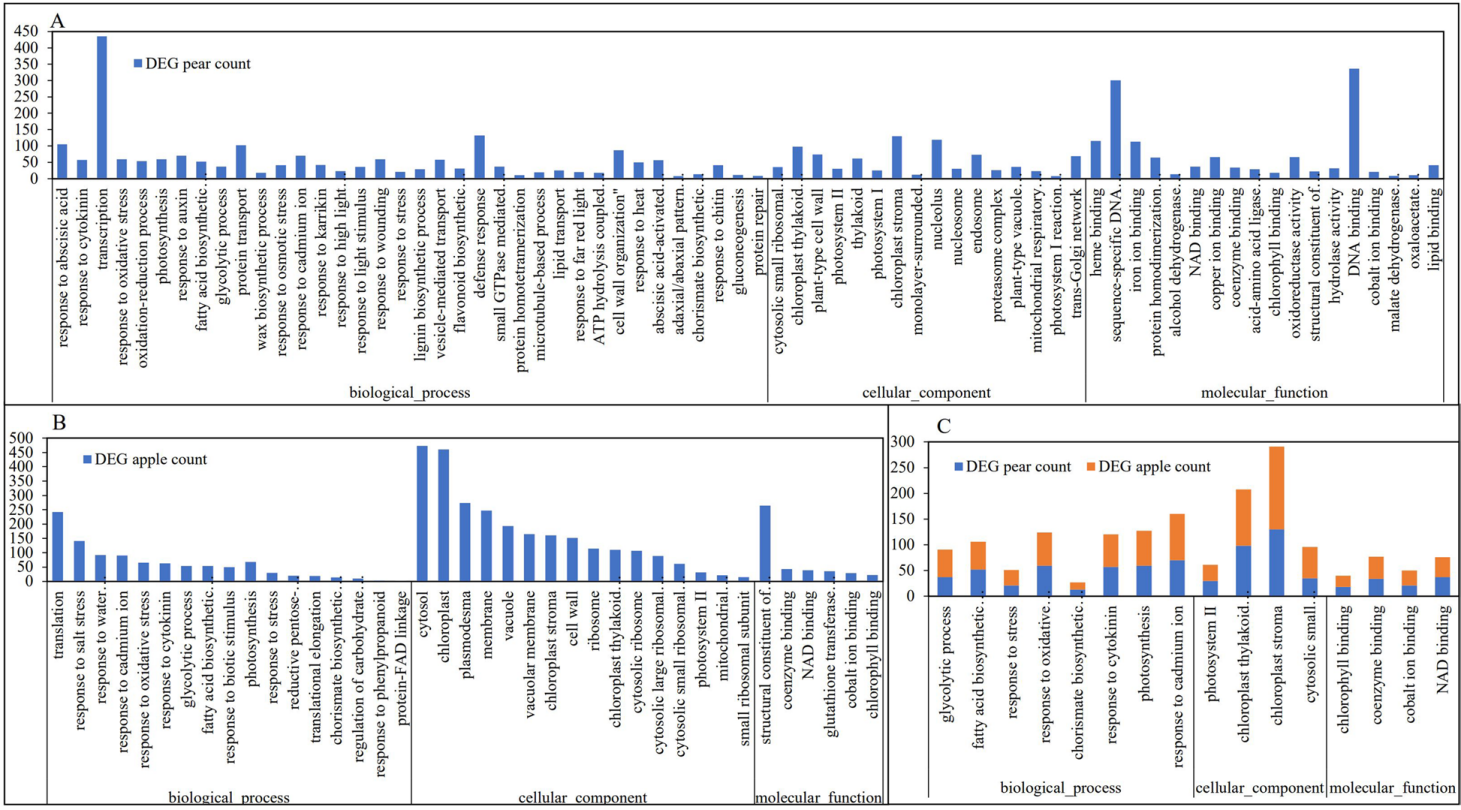
Functional enrichment of all differentially expressed genes with –log (*p*-value) > 6. 36 biological pathways, 16 cell components and 18 molecular functions in pear (**A**), 17 biological pathways, 16 cell components and 6 molecular functions in apple (**B**). 8 biological pathways, 4 cell components and 4 molecular functions (**C**) both enriched to pear and apple

GO enrichment analysis was carried out on 5921 and 5744 different expression genes in pear and apple respectively, of which 4332 and 4378 genes could be annotated to 3261 and 3190 GO term respectively. GO functional enrichment accorded with a hypergeometric distribution, in which *P* < 0.05 was significantly enriched to 855 and 533 terms (Supplementary Fig S1) in pear and apple respectively. GO terms with -log (*P*-value) > 6 were further enriched to 36 biological pathways, 16 cell components and 18 molecular functions in pear different expression genes (Fig. 5A), 17 biological pathways, 16 cell components and 6 molecular functions in apple different expression genes (Fig. 5B). The common GO term with -log (*P*-value) > 6 in pear and apple different expression genes were enriched into 8 biological pathways, 4 cell components and 4 molecular functions (Fig. 5C). The main enriched terms in biological processes were glycolytic process, fatty acid biosynthetic process, response to stress, response to oxidative stress, chorismate biosynthetic process, response to cytokinin, photosynthesis and response to cadmium ion (Fig. 5C).

### Differential expression genes pathway enrichment analysis of pear and apple at three stages

**Fig. 6:**
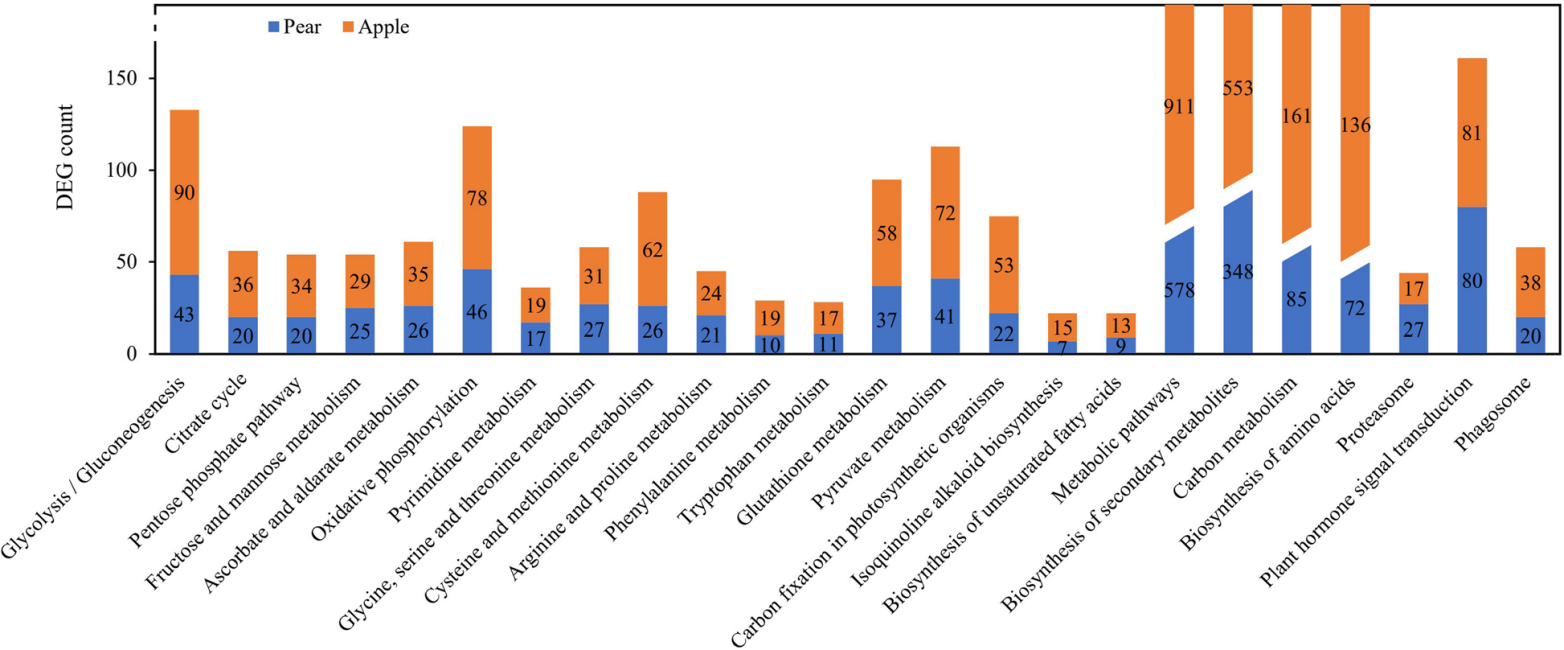
KEGG pathway enrichment both in pear and apple differential expression genes with *P* < 0.05. The ordinate represents the common 24 KEGG pathways

The Kyoto encyclopedia of genes and genomes (KEGG) pathway can help to further determine biological functions and interactions of genes [30]. Based on a comparison against the KEGG database, of the 40,402 genes in pear, 13,102 (32.43%) genes had significant matches and were assigned to 133 KEGG pathways; of the 56,781 genes in apple, 18,459 (32.51%) genes had significant matches and were assigned to 134 KEGG pathways. Among the 5921 different expression genes in pear, 1929 genes were assigned to 123 KEGG pathways, and 50 pathways with *P* < 0.05 were significantly enrichment (Supplementary Fig S2). Among the 5744 apple different expression genes, 2578 genes participated in 128 pathways, and 30 pathways with *P* < 0.05 were significantly enrichment (Supplementary Fig S3). 24 pathways were significantly enrichment both in pear and apple, which mainly involved in Metabolic pathways (578 genes in pear, 911 genes in apple), Biosynthesis of secondary metabolites (348 genes in pear, 553 genes in apple), Carbon metabolism (85 genes in pear, 161 genes in apple) and Biosynthesis of amino acids (72 genes in pear, 136 genes in apple) etc. (Fig. 6).

### Co-expression modules analysis of differential expression genes of pear and apple at three stages

**Fig. 7:**
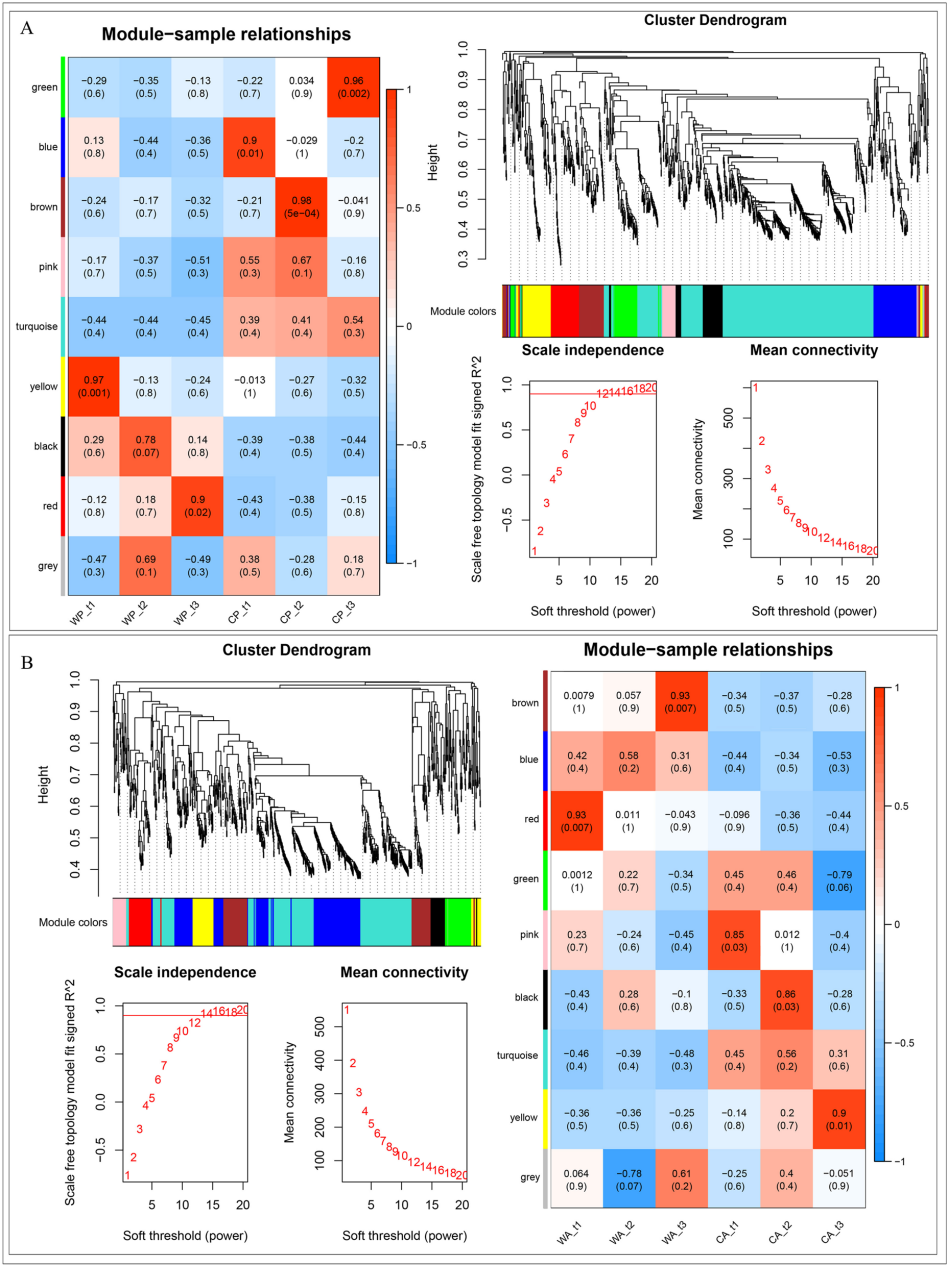
Co-expression module of 1068 and 969 co-differential expression genes at three stages of pear (**A**) and apple (**B**) respectively. Screening of soft-threshold power as 14 at *R*^2^ = 0.8(red line), which divided genes into 9 color modules. Correlation between each module and six samples showed with the correlation coefficient and *P* value, red indicates positive correlation and blue means negative correlation

1068 and 969 genes expressed differently through all development stages in pear and apple respectively. According to the correlation between gene expression, these 1068 and 969 genes been divided into 9 models respectively using WGCNA with Power = 14 (Fig. 7) [31]. The grey module, which is a non-functional module, has 4 and 2 genes in pear and apple respectively. The correlations between modules and six samples were examined. Most of the 9 modules were negatively correlated with each other in six samples, but the positive correlation was especially significant at certain stages (*P* < 0.05). Function classification indicated that yellow in WP_t1 (*r* = 0.97, 84 genes) associated with flavonoid biosynthetic process, response to cadmium ion etc.; red in WP_t3 (*r* = 0.90, 78 genes) response to transcription, high light intensity, heat, salt stress and abscisic acid; blue in CP_t1 (*r* = 0.90, 112 genes) associated with transcription, glycolytic process, photosynthesis; brown in CP_t2 (*r* = 0.98, 85 genes) also focuses on response to water deprivation, abscisic acid, cold and salt stress; green in CP_t3 (*r* = 0.96, 78 gens) focus on plant-type cell wall organization, malate metabolic process etc. (Fig. 7A, Additional file 1). Module red in WA_t1 (*r* = 0.93, 62 gens) associated with flavonoid biosynthetic process, anthocyanin-containing compound biosynthetic process, response to stress; brown in WA_t3 (*r* = 0.93, 117 genes) associated with transcription, defense response, response to cold; pink in CA_t1 (*r* = 0.85, 35 genes) associated with response to light stimulus, ATP hydrolysis coupled proton transport; black in CA_t2 (*r* = 0.86, 41 genes) associated with response to response to water deprivation, photosynthesis, response to cytokinin; yellow in CA_t3 (*r* = 0.90, 72 genes) associated with translation, defense response, fatty acid biosynthetic process (Fig. 7B, Additional file 1). Combined with the gene interactions within each module, 10 key genes significantly expressed in each module of pear and apple were selected to conduct the expression level analysis (Additional file 2, Supplementary Fig S4).

## Discussion

In this study, we reported on the next-generation sequencing study of the temporal and spatial mRNA expression profiling of pear and apple fruit trees. A differentially expression genes (DEG) approach with functional classification enabled identifying those mechanisms related to long periods of domestication between wild and cultivated materials [32]. STEM, positive and negative correlation analysis were applied in integrated analysis of DEGs data [29]. WGCNA clusters differential expression genes into modules and correlates them with phenotypes [31]. These provided a molecular basis underlying the differences of wild and cultivated materials.

### Reliability of sample data

Studies have shown that gene expression has biological variability among different individuals, and the variable degree of expression varies among different genes, while transcriptome sequencing technology can not eliminate this variability [33, 34]. At present, the most common and effective method is to set up biological duplication in experimental design. Although there was no biological duplication for individual specie in this study, we analyzed the correlation of the same gene expression of pear data in this study with published data [14] by comparing the same fruit development stages, and correlation coefficient reached 0.95, 0.96 and 1 (*P* < 2.2e-16) (Supplementary Fig S5). At the same time, the pear and apple species combined as a repeat to jointly analyze the differences between wild and cultivated fruits during domestication which strongly revealed the consistent process of domestication in *Rosaceae* fruit trees.

### Temporal and spatial growth patterns of pear and apple fruits

The young fruit, expanding and maturity stages are involved in growth phases of fruits and established with phenological period of different area in previous study [14, 35]. To determine transcriptional regulation changes that occurred among wild and cultivar fruits during different growth stages, we analyzed the differential expression genes. In generally, the number of differential expression genes could represent different growth degree, the greater number between species, the larger difference between them [36]. During the growth of fruit, the number of differentially expressed genes between wild and cultivated fruit almost become more, and the difference between young fruit stage and maturity stage is the biggest both in pear and apple (Fig. 3). We also found that common differential expression genes were not significantly associated with wild fruit growth at expanding stage both in pear (*r* = 0.78, *P* = 0.07) and apple (*r* = 0.58, *P* = 0.2) (Fig. 7). Compared with others, the number of differential expression genes in wild and cultivar apple at expanding stages was smaller, it may be due to some internal mechanism in the apple that there was little change at this period.

### GO and pathways involved in phenotypes and intrinsic qualities of wild and cultivated fruits

During the long period of domestication from wild fruit to cultivated fruit, the external and internal qualities changed greatly and the data in this study confirm these changes (Figs. 1, 2 and Table 3). The GO and pathways analysis revealed that differential expression genes both in pear and apple involved in glycolytic process, fatty acid biosynthetic process, response to stress, oxidative stress, cytokinin, cadmium ion, photosynthesis, metabolic pathways and signal transduction played major role in domestication of fruits (Figs. 5 and 6).

In this study, fruit size and weight differences were the most obvious feature in the comparison of wild and cultivar materials. Studies have generally believed that they usually determined by the number of cells, cell volume and intercellular space [15, 37]. Daccord et al. found that in two apple lines, the cell layer which has larger fruit was significantly larger than the another one [15]. Plant endogenous hormones as well as environment factors can regulate progression of plant growth and development through the cell cycle [36]. Several plant hormones, in particular auxin, cytokinin, ethylene and gibberellins are known to regulate cambial development and cell enlargement [38–40]. In the present study, some differential expression genes involved in ethylene biosynthetic process, response to cytokinin, abscisic acid were identified (Additional file 1). This suggests that cell division is essential to rapid volume enlargement of fruits during their early developmental stages.

Photosynthesis is the process by which plants use chlorophyll to convert carbon dioxide and water into organic matter and it plays a key role in plant growth and sugar accumulation [41, 42]. In fact, a common trend both on pear and apple was found that total sugar content of cultivated variety was significantly higher than that of wild and increases during fruit growth, while organic acids showed the opposite trend, these findings are consistent with previous studies [27, 28]. Some studies have suggested that up-regulation of plasma membrane aquaporins improves the photosynthetic activity and growth of trees [43]. Our study also found the cellular component such as photosystem II, ribosomal and molecular function such as chlorophyll binding, coenzyme binding, NAD binding were significantly enriched both in wild cultivar pear and apple differential expression genes (Fig. 5), these results all work together and indicate that photosynthetic capacity has an important effect on the differentiation of wild and cultivated fruit trees. On the other hand, chlorophyll binding and photosynthesis determine the change in peel color [44]. The formation of different colors of fruit is caused by pigments, the pigments in apples are chlorophyll, anthocyanin and carotenoid, which form green, red and yellow respectively [45]. In addition, it was found that differences in light spectrum and intensity had effects on the formation of pigments [46]. In the present study, peel color regulation mainly involved in leucoanthocyanidin reductase activity and flavonoid biosynthetic process, several related genes which have been reported in previous study were up-regulation or down-regulation in apple (Supplementary Fig S6) and they interact to regulate peel color [12, 47, 48].

Some studies reported that wild materials have more advantages in the face of adversity stress or some external condition, and sometimes key genes were selected from them to develop new resistant varieties [10, 49, 50]. Many genes involved in response to stimulus have been reported in the development of rice and sweet orange [50, 51]. In this study, some differential expression genes involved in defense response to stimulus, such as oxidative stress, cytokinin, auxin or abscisic acid stimulus, cadmium ion, cold, wounding, light were detected during the growth of fruits, indicting the important role of environment factors in fruit’s domestication. In addition, there are excellent resistance genes in wild species, which need further study. In the development of domestication from wild to cultivar fruit, the dominant metabolic pathways included biosynthesis of secondary metabolites, carbon metabolism, glycolysis / gluconeogenesis, biosynthesis of amino acids (Fig. 6) have been determined that among the most regulated during developing stages of plants. These metabolic processes can provide the energy and components for DNA replication, translation, signal transduction, hormone biosynthesis and cellular growth [36, 52], which are essential for the rapid domestication of fruits under natural conditions.

## Materials and methods

### Plant material and biological measurements

The wild pear ‘Douli’ (*P. calleryana* Decne) and cultivar pear ‘Dangshansuli’ (*P. bretschneideri* Rehd) were collected from the experimental farm of Nanjing Agricultural University. The wild apple ‘Xifuhaitang’ (*M. micromalus* Makino) and cultivar apple ‘Golden Delicious’ (*M. domestica*) were collected from Zhenzhou Fruit Research Institute, Chinese Academy of Agricultural Sciences (ZFRI, CAAS). Pear fruits were harvested on May 6^th^, June 30^th^ and September 6^th^ 2014; Apple Fruit were harvested on May 6^th^, June 6^th^ and September 17^th^ 2014, and which corresponded to the young fruit stage, expansion stage and maturity stage respectively (Table 1). Ten fruits were picked from each stage. The fruits in the same group were cut into small pieces, pooled and packaged in the field, and immediately frozen in liquid nitrogen and then samples were stored in -80 °C until RNA extraction. In addition, the single fruit weight was measured by electronic balance in another ten fruits, the vertical diameter and horizontal diameter of each fruit was measured by vernier caliper on the cross section of the center of the fruit. The measurement of soluble sugar (sorbitol + fructose + glucose + sucrose) and organic acids (quinic acid + citric acid + malic acid + oxalic acid + shikimic acid) was performed by high performance liquid chromatography (HPLC) according to the classical methods as described with slight modification [53]. Five grams of fruit tissue were ground into powder with liquid nitrogen in a mortar and pestle. After mixed with 6 ml 80% ethanol and placed into a 37 °C bath for 30 min. The mixture was centrifuged at 15,000 g for 15 min at 4 °C. The above steps were repeated three times to make sure all sugars and acids were extracted for a total volume of supernatants of 50 mL. The supernatant was recovered and immediately filtered through a SEP-C18 column (Waters, WAT020515) to eliminate any interfering apolar residues and through a 0.45 μm Sep-Pak filter (Jasco France, TR200102) to eliminate large particles. The extract was then ready for HPLC system of sugar and acid contents measurement following the characteristics described by Yao et al. (2010) and Sha et al. (2011) respectively [27, 28]. Sample contents were established using external standards and expressed in milligram per gram fresh weight (FW).

### Library preparation for transcriptome sequencing

The fruit tissues from 12 samples (three development stages, four individuals) were collected for RNA preparation. Total RNA was extracted using a TRIzol reagent (Gibco BRL) following the manufacturer’s instructions and mRNA was purified from total RNA using poly-T oligo-attached magnetic beads. RNA concentration and purity were measured using NanoDrop 2000 (Thermo Fisher Scientific, Wilmington, DE). After fragmentation, first strand cDNA was synthesized using random hexamer primer and second strand cDNA synthesis was subsequently performed using DNA Polymerase I and RNase H. In order to select cDNA fragments of preferentially 240 bp in length, the library fragments were purified with AMPure XP system (Beckman Coulter, Beverly, USA). After PCR amplification of the selected fragment, library quality was assessed on the Agilent Bioanalyzer 2100 system and then sequenced with Illumina platform and paired-end reads were generated. The raw data of the experiment are submitted to Sequence Read Archive (SRA) of NCBI under accession number SRR9291270, SRR9291271, SRR9891658 and SRR9891659.

### Transcriptome data analysis

Raw reads of fastq format were firstly processed through in-house perl scripts. In this step, the adaptor sequences and low-quality sequence reads were removed from the data sets. Raw sequences were transformed into clean reads after data processing. These clean reads were then mapped to the pear and apple reference genome sequence respectively by Tophat tool soft (http://ccb.jhu.edu/software/tophat/index.shtml) [14, 17]. Only reads with a perfect match or one mismatch were further analyzed and annotated based on the reference genome. Gene function was annotated based on the following databases: Nr (NCBI non-redundant protein sequences); Nt (NCBI non-redundant nucleotide sequences); Pfam (Protein family); KOG/COG (Clusters of Orthologous Groups of proteins); Swiss-Prot (A manually annotated and reviewed protein sequence database); KO (KEGG Ortholog database); GO (Gene Ontology). Gene expression levels were estimated by transcripts per million (TPM) using Kallisto software (https://pachterlab.github.io/kallisto/).

### Differential expression analysis

The differential expression transcripts and genes in fruits were analyzed by R-package DEGseq [32]. DEGseq is more efficient in the detection of differentially expressed genes directly by using the number of reading segments from transcripts, and in the detection of the MA map of differentially expressed genes by mapping the log_2_ value of reading segments from a particular transcript. It is assumed that the number of reads to a specific gene in two different samples is C_1_ and C_2_ respectively: M = log_2_C_1_ – log_2_C_2_, A = (log_2_C_1_ + log_2_C_2_)/2. *P* value < 0.001 and |Fold change|> 2 was set as the threshold for significantly differential expression.

### GO and KEGG pathway enrichment analysis of differential expression genes

Gene Ontology (GO) is an international web site for functional annotations of genes, providing a range of semantics for describing the characteristics of genes and gene products, including cellular components, molecular functions and biological pathways (http://www.geneontology.org/). Kyoto Encyclopedia of Genes and Genomes (KEGG) a database resource for understanding high-level functions and utilities of the biological system, such as the cell, the organism and the ecosystem, from molecular-level information, especially large-scale molecular datasets generated by genome sequencing and other high-throughput experimental technologies (http://www.kegg.jp/). Gene Ontology and KEGG pathway enrichment analysis of the differential expression genes were implemented by R script based non-central hyper-geometric distribution with *P* value < 0.05 [54].

### Short time series expression analysis of differential expression genes

Short Time-series Expression Miner (STEM) [29] is a software for sequence clustering, comparison and visual expression in a short time. It is suitable for 3–8 time points. The maximum number of model profiles is 50, the maximum unit change in model profiles between time points is 2. STEM can order profiles by ‘Swap rows and columns’, ‘Profile ID’, ‘Significance’ and ‘Correlation’. In this study, all differential expression genes of four individuals (wild and cultivar pear, wild and cultivar apple) as input file, and regulation trend significantly changed with *P* value < 0.05.

### Differential expression genes co-expression module recognition

The R package of WGCNA (Weighted Gene Co-Expression Network Analysis) is a common tool for weighted correlation network analysis [31]. In this study, 1068 and 969 common differential expression genes at three stages in pear and apple as input data respectively. All data were complete and no outliers. The soft threshold power was screened and the gene was divided into nine modules by selecting power as 14. Modules are defined as clusters of densely interconnected genes. Finally, the correlation among each modules and phenotypes at three development stages were calculated, the significantly correlated modules with *r* > 0.8 and *P* value < 0.05.

## Supplementary Information

**Additional file 1.**

**Additional file 2.**

**Additional file 3.**

## Data Availability

Raw reads of the experiment are submitted to NCBI SRA database (https://www.ncbi.nlm.nih.gov/sra), the accession number of the study is SRR9291270, SRR9291271, SRR9891658 and SRR9891659.
