# Supplementary material for: Study on the differences of gene expression between pear and apple wild cultivation materials based on RNA-seq technique
Source: BMC Plant Biol. 2021 Jun 4;21:256. doi: 10.1186/s12870-021-03051-0 (PMC8176607; doi:10.1186/s12870-021-03051-0)
Supplement: Supplementary file 3 — Additional file 3. [file 12870_2021_3051_MOESM3_ESM.pdf]

STUDY ON THE DIFFERENCES OF GENE EXPRESSION BETWEEN PEAR AND APPLE WILD CULTIVATION MATERIALS BASED ON RNA-SEQ TECHNIQUE

Huangwei Zhang#, Meng Li#, Min Kong, Jim M Dunwell, Yuyan Zhang, Chao Yue, Juyou Wu, Shaoling Zhang

Supplementary Figure

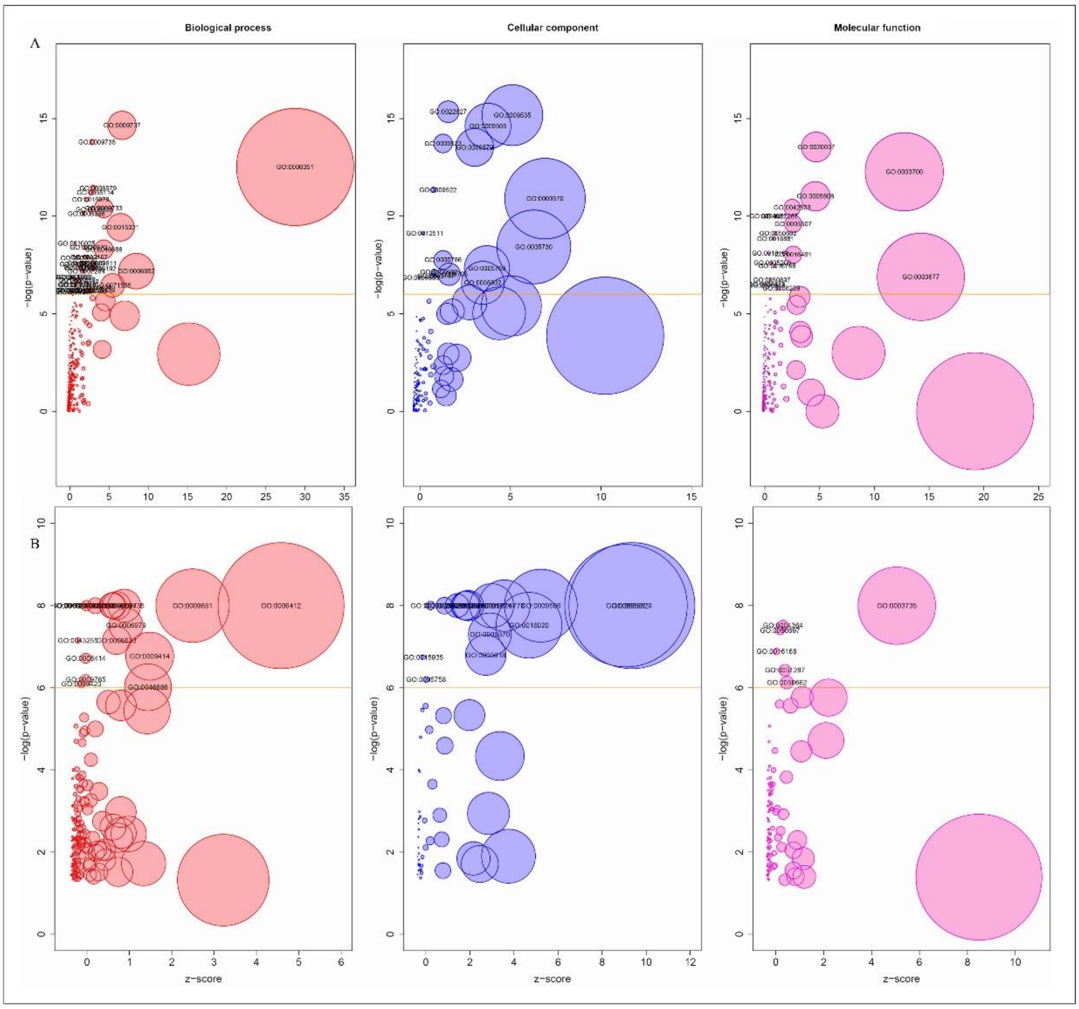

Fig S1 Functional enrichment of all differentially expressed genes with *P* <0.05 in pear (A) and apple (B). From left to right are the biological process, cell components and molecular functions, respectively. The ordinate –log (*P*-value) represents the probability of GO term enrichment, and the yellow line is 6. The P-value is equal to $p = 1 - \sum_{i=0}^{m-1} \frac{\frac{M}{i} \times \frac{N-M}{n-i}}{\frac{N}{n}}$, N: the total number of genes annotated by GO, M: the number of genes belonging to a GO subclass, m: The number of differential gene sets for GO enrichment analysis, i: the number of M in m, The abscissa Z-score: the distance between the number of GO term and the mean (positive or negative). Z-score = (the number of a GO term − the mean of GO terms)/ standard deviation. Circle size represents the number of genes enriched by a GO term.

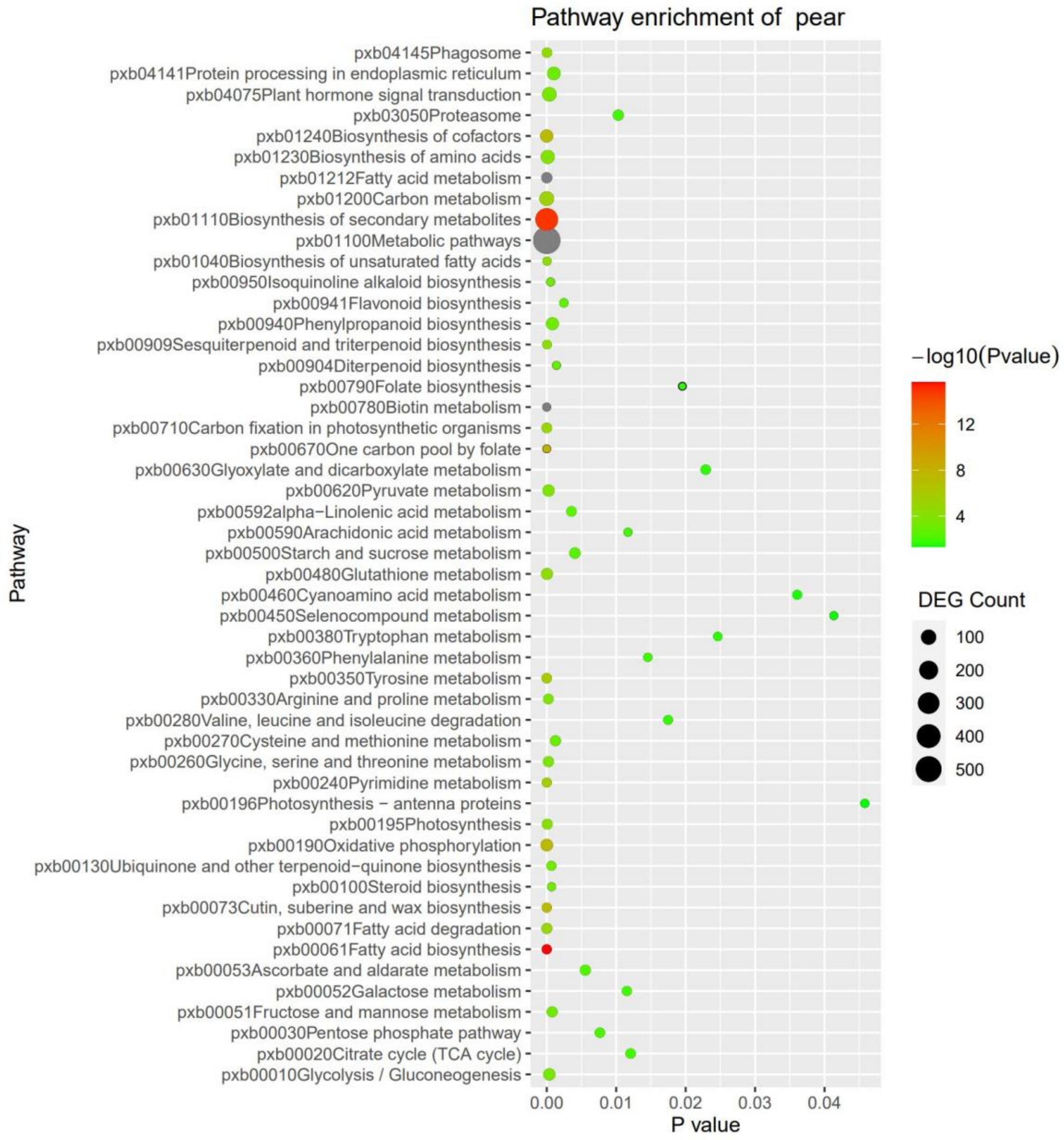

Fig S2 Fifty pathways with $P < 0.05$ were significantly enrichment in 5921 different expression genes of pear. Colors represent -log10 (P value) significant level from green to red and the circle size means the number of genes enriched by a pathway.

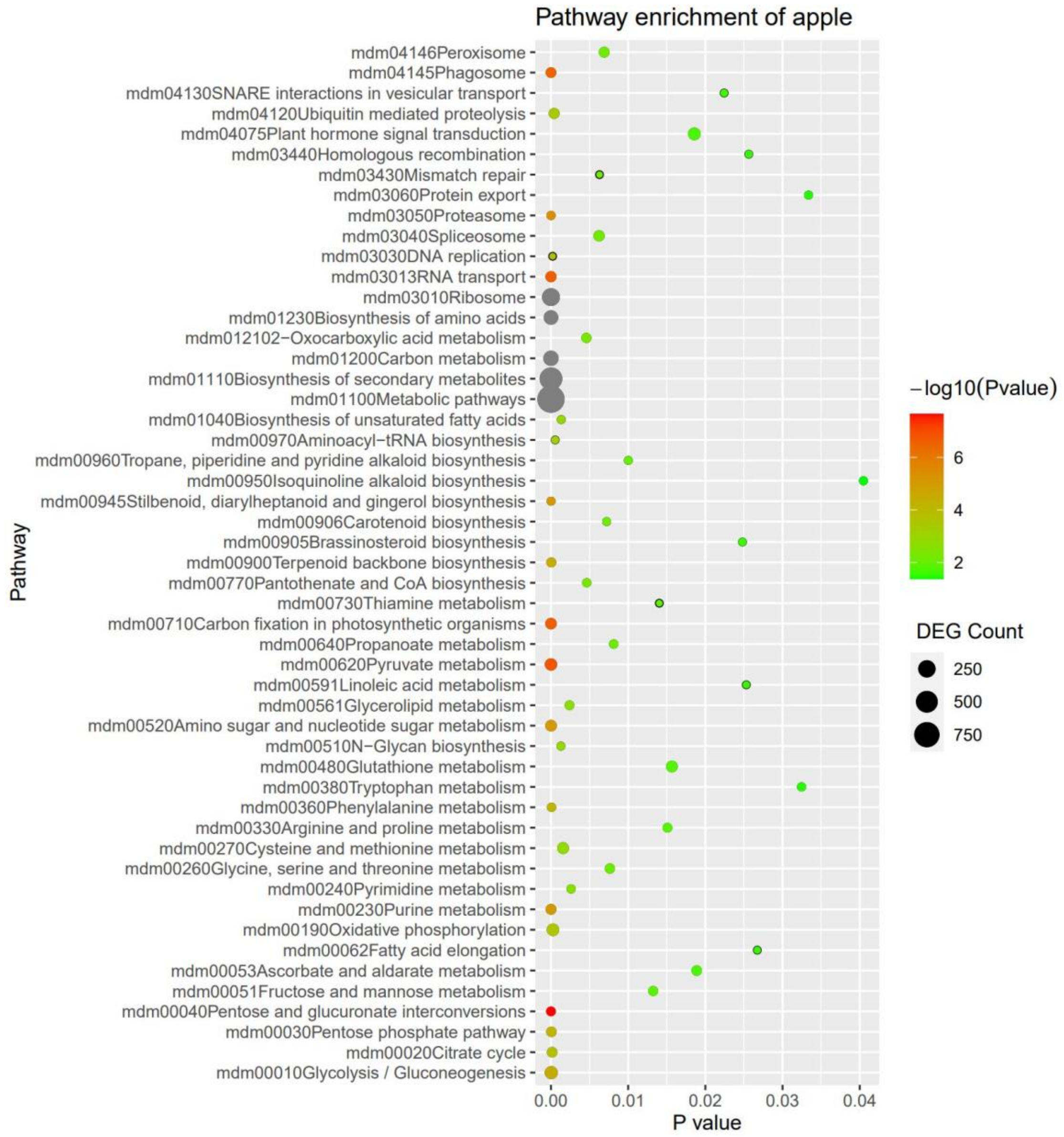

Fig S3 Thirty pathways with *P* < 0.05 were significantly enrichment in 5744 different expression genes of apple. Colors represent -log10 (*P* value) significant level from green to red and the circle size means the number of genes enriched by a pathway.

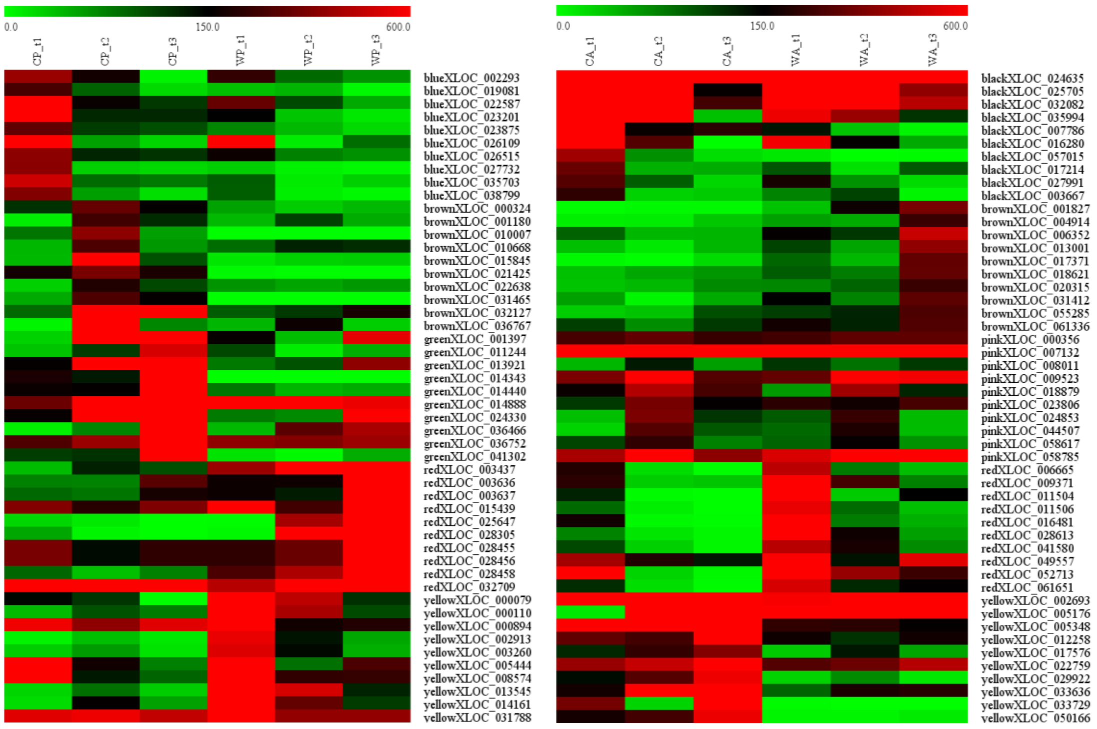

Fig S4 Heat map of top 10 hub gene expression in phenotypic significantly related modules of pear (left) and apple (right). Module blue, brown, green, red and yellow in pear associated with CP_t1, CP_t2, CP_t3, WP_t3 and WP_t1 respectively. Module black, brown, pink, red and yellow in apple associated with CA_t2, WA_t3, CA_t1, WA_t1 and CA_t3 respectively.

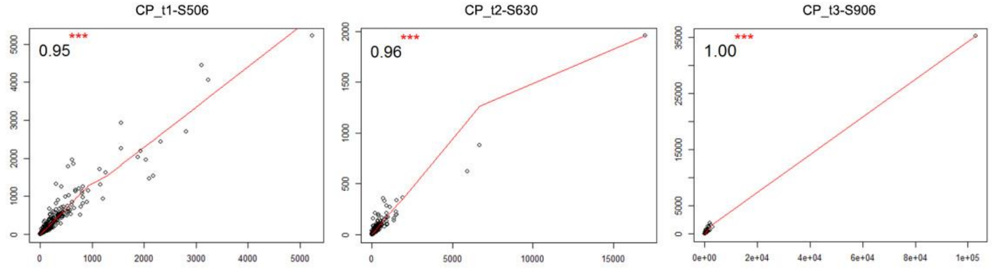

Fig S5 Correlation between pear transcriptome data in this study and published data. X-axis and Y-axis shows the expression level of the same genes (TPM). From left to right are scatter plots of gene expression at young fruit stage, expansion stage and mature stage (with fitting lines) respectively. CP_t1, CP_t2 and CP_t3 were collected on May 6th, June 30th and September 6th respectively in this study, S506, S630 and S906 were transformed from published gene expression data. The top left corner shows the correlation coefficient, asterisk (*) representing the significance level, *** means $P < 0.001$.

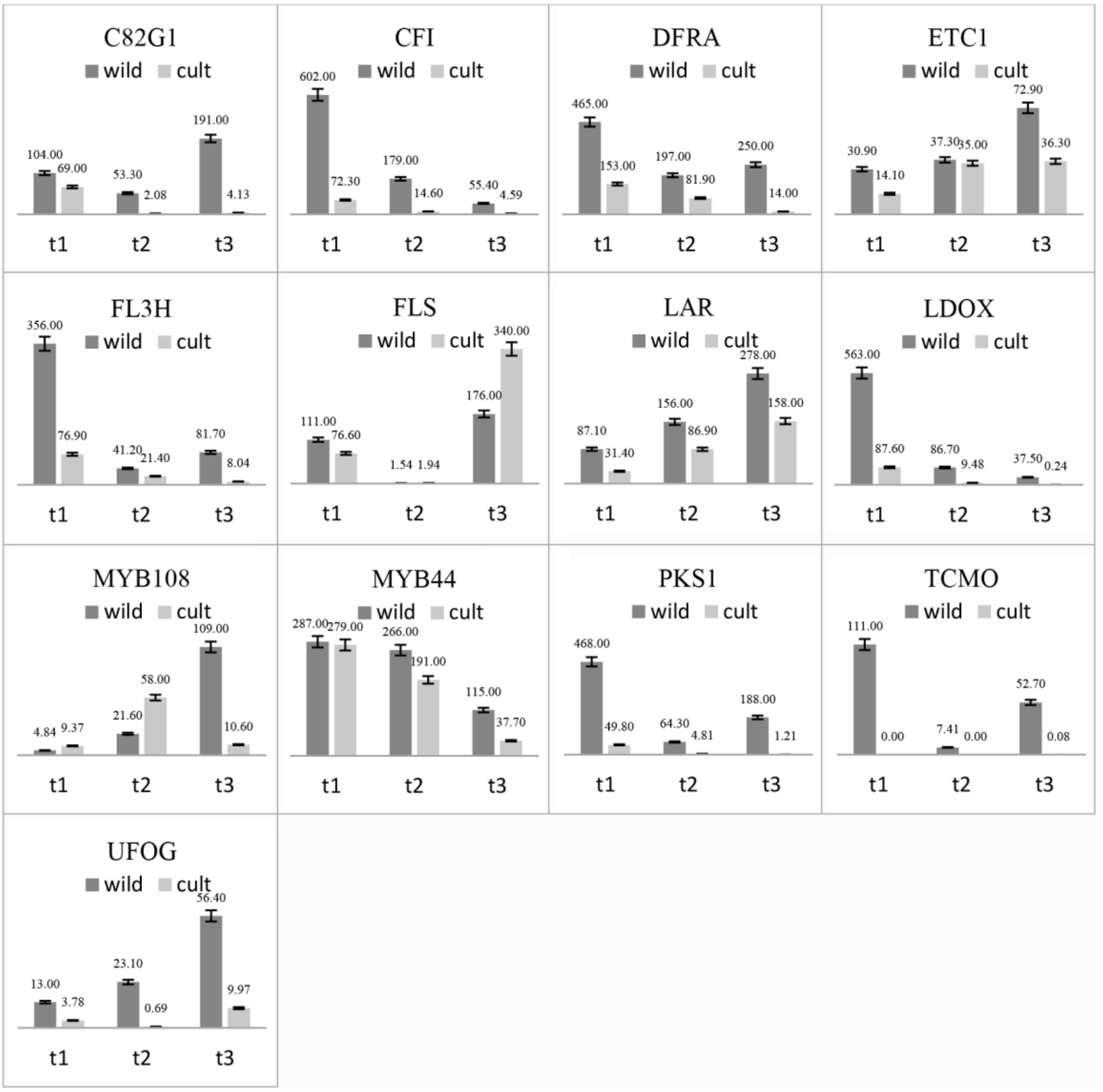

Fig S6 Transcriptional expression of color-related key genes in apple (TPM). Wild apple 'Xifuhaitang', cultivar apple 'Golden Delicious'; *C82G1*: Cytochrome P450 82G1, *CFI*: Chalcone-flavonone isomerase, *DFRA*: dihydroflavonol 4-reductase/flavanone 4-reductase, *ETC1*: MYB-like transcription factor ETC1, *FL3H*: Naringenin,2-oxoglutarate 3-dioxygenase, *FLS*: Flavonol synthase/flavanone 3 hydroxylase, *LAR*: Leucoanthocyanidin reductase, *LDOX*: Leucoanthocyanidin dioxygenase, *MY108*: Transcription factor MYB108, *MYB44*: Transcription factor MYB44, *PKS1*: Polyketide synthase 1, *TCMO*: Trans-cinnamate 4-monooxygenase, *UFOG3*: UDP-glucose flavonoid 3 Oglucosyltransferase 3.
